# A study of gene expression programming algorithm for dynamically adjusting the parameters of genetic operators

**DOI:** 10.1371/journal.pone.0321711

**Published:** 2025-06-02

**Authors:** Kejia Liu, Yiping Teng, Fang Liu

**Affiliations:** School of Computer Science, Shenyang Aerospace University, Shenyang, China; Sichuan University, CHINA

## Abstract

The fast developments in artificial intelligence together with evolutionary algorithms have not solved all the difficulties that Gene Expression Programming (GEP) encounters when maintaining population diversity and preventing premature convergence. Its restrictions block GEP from successfully handling high-dimensional along with complex optimization problems. This research develops Dynamic Gene Expression Programming (DGEP) as an algorithm to control genetic operators dynamically thus achieving improved global search with increased population diversity. The approach operates with two unique operators which include Adaptive Regeneration Operator (DGEP-R) and Dynamically Adjusted Mutation Operator (DGEP-M) to preserve diversity while maintaining exploration-exploitation balance during evolutionary search. An extensive evaluation of DGEP occurred through symbolic regression problem tests. The study employed traditional benchmark functions and conducted evaluations versus baselines Standard GEP, NMO-SARA, and MS-GEP-A to assess fitness outcomes, R² values, population diversification, and the avoidance of local optima. All key metric evaluations showed that DGEP beat standard GEP along with alternative improved variants. DGEP produced the optimal results for 8 benchmark functions that produced 15.7% better R² scores along with 2.3 × larger population diversity. The escape rate from local optima within DGEP reached 35% higher than what standard GEP could achieve. The DGEP model serves to enhance GEP performance through the effective maintenance of diversity and improved global search functions. The results indicate that adaptive genetic methods strengthen evolutionary procedures for solving complex problems effectively.

## 1. Introduction

Gene Expression Programming (GEP) [1,2] functions as an effective adaptive evolutionary algorithm because it applies unique genotype-to-phenotype mappings to address optimization problems characterized by high nonlinearity and multimodality with efficiency [3–5]. GEP demonstrates its strength by offering efficient optimization strategies with highly interpretable models when solving problems in industrial process optimization [6–8] and intelligent manufacturing [9,10] as well as financial market prediction [11]. The evolution encounters substantial issues that affect the performance of GEP algorithms despite their effective properties. The reduction of population variety combined with deteriorating global navigation ability makes GEP exhibit premature local optimization which reduces its effectiveness for complex real-world problems.

A wide range of improvement techniques for population diversity and search performance has been developed to advance GEP throughout recent years. Experts who focus on enhancing classic genetic operators [12–15] have developed adaptive system controls to modify operator parameters during evolution improving the exploration-exploitation equilibrium. The prevention of premature convergence combined with improved global searching capabilities is achieved through multiple diversity-maintaining strategies described in [16,17]. GEP achieves its best results when integrated with reinforcement learning and particle swarm optimization in addition to similar techniques for optimizing high-dimensional complex search spaces according to research reports [18–20]. Multi-objective GEP methods [15,21] also allow for the simultaneous optimization of conflicting objectives, expanding the applicability of GEP to more complex problem domains.

These methods have made significant progress in improving the performance of GEP, however, they still fail to overcome some fundamental issues. First, in the GEP algorithm, all offspring genotypes are directly or indirectly derived from the initial population. Although genetic operations such as mutation, crossover, and recombination introduce diversity into the population, once the genotypes in the evolutionary process become homogeneous, these methods struggle to effectively restore population diversity. Second, some methods attempt to adjust parameters based on fixed rules or predefined conditions, but they often lack the flexibility needed to dynamically adapt to the varying demands at different stages of evolution.

To overcome the limitations of existing GEP algorithms, this study introduces a novel approach, Dynamic Gene Expression Programming (DGEP), which introduces two genetic operators proposed in this paper: the Adaptive Regeneration Operator (DGEP-R) and the Dynamically Adjusted Mutation Operator (DGEP-M). This research developed novel operators that aim to solve traditional GEP weaknesses regarding global search and population diversity. We established DGEP-R to work during individual selection phases whereas DGEP-M operated after selection in genetic operations and maintained a supportive relation between the two operators. DGEP-R operates at evolutionary turning points to add new potential solutions that help increase diversity as well as exploration space during stagnant fitness phases. DGEPM regulates mutation rates according to the evolutionary development dynamics. The dynamic genetic operator mechanism of DGEP-M promotes fitness-based control of mutation frequency which creates a balanced search method that protects elite solutions but maintains population variety. Experimental tests on symbolic problems have verified that DGEP leads to superior performance compared to standard GEP and alternative advanced variants of GEP. DGEP demonstrated superior solution accuracy and diverse population maintenance while strengthening its ability to explore global search spaces according to experimental results. Our main work achievements consist of the following:

1. To enhance population diversity, we proposed an Adaptive Regeneration Operator (DGEP-R) by introducing new individuals at critical evolutionary stages and when fitness stagnates, achieving diversity maintenance, premature convergence prevention, and significant improvement of global search capability.
2. To balance exploration and exploitation, we developed a Dynamically Adjusted Mutation Operator (DGEP-M) that modulates the mutation rate based on evolutionary progress.
3. Experiments on symbolic regression problems show that the proposed DGEP algorithm outperforms traditional and improved GEP algorithms in solution accuracy, population diversity, and global search capability.

The Genetic Expression Programming methodology serves as a widely accepted solution for optimizing complex problems and symbol-based regressive modeling. The primary difficult with standard GEP algorithms involves deteriorating population diversity during evolution that generates early convergence thus limiting the algorithm’s global space exploration. Over time genetic material within the population collects similar traits which prevents the discovery of optimal solutions.

GEP optimization receives enhancements through adaptive genetic operators while population control mechanisms and hybrid system optimization strategies work to tackle this issue. The methods also introduce performance problems when implemented in complex dynamic environments because they use fixed operational parameters and create extra processing requirements. This paper presents the Dynamic Gene Expression Programming (DGEP) algorithm that incorporates two innovative operators: Adaptive Regeneration Operator (DGEP-R) and Dynamically Adjusted Mutation Operator (DGEP-M). The operators use continuous fitness assessment of the population together with evolutionary monitoring to dynamically control genetic operations which both conserve diversity and boost exploration abilities as well as convergence speed. The dynamic adjustment of genetic parameters through DGEP improves both the GEP search ability and prevents premature convergence thus resulting in improved optimizers.

The second part of this work explores related studies which have improved the GEP algorithm. The third part defines the problem statement and presents the necessary background information. Section 4 details the implementation structure of the proposed DGEP algorithm. Section 5compares DGEP with standard GEP and other improved algorithms through experiments and analysis. Section 6 summarizes this work and presents future work.

## 2. Related work

Modern Gene Expression Programming research enables increased efficiency by following three major directions which involve: advanced genetic operator optimization and population diversity management as well as hybrid and multi-objective integration techniques.

### 2.1 Improving genetic operators

Research has actively worked to enhance standard GEP genetic operators including mutation crossover and recombination through several published works [14,15,17,22,23] to improve population diversity while stopping premature convergence. Evolutionary algorithms need these operators to perform exploration and exploitation operations harmoniously [9,24–26]. The evolutionary population state serves as a basis for adaptive genetic operator parameters [12,13,27,28] adjustment in implementations which include [10,11,29]. The evolutionary stage maintains an equilibrium between exploratory and exploitative traits because of this mechanism.

### 2.2 Population diversity maintenance mechanisms

The research field of GEP now emphasizes sustaining population diversity during evolution. The implementation of different diversity preservation approaches through new selection methods is being researched. The Clonal Selection Algorithm (CLONALG) [30] draws its ideas from the immune system by applying data-like antigens. The algorithm shows better computational power and expression ability than conventional GEP approaches. MS-GEP-I manual intervention strategy actively implements outside measures when population diversity deteriorates or evolutionary stagnation happens [13]. For instance, when no improvement in fitness is observed over several generations, individuals are randomly replaced, or the process reverts to parent or grandparent generations to alter the evolutionary trajectory and reinvigorate the search process. In addition, dynamic population generation (DPGP) [12] adjusts the population size dynamically during evolution to prevent the algorithm from becoming trapped in local optima and to maintain diversity. Modern engineering optimization problems have received enhancements through newly developed meta-heuristic algorithms in recent years. The Artificial Lemming Algorithm (ALA) uses Lemming’s natural behaviors to resolve actual engineering optimization obstacles [31]. Multi-Strategy Boosted Snow Ablation Optimizer (MSAO) represents an enhanced version of Snow Ablation Optimizer through its integration of multiple strategies which produces superior global optimization performance [32,33]. The development of improved algorithms reflects the continuous research to enhance both the efficiency of evolutionary computation along its solution outcomes.

The field of genetic programming now benefits from newly developed adaptive processes together with hybrid optimization methods that enhance the speed of evolutionary search operations. Scientists have analyzed self-adaptive resource allocation in cloud computing as shown by [to prove how adaptable resource distribution optimizes operational efficiency. The wide range of real-world problems has been efficiently tackled by evolutionary algorithms in software engineering [34] while they also lead to improved solutions in manufacturing optimization [3] and predictive modeling [35]. Evolutionary approaches for genetic programming identified as multi-objective optimization [35] enabled better management between exploration and exploitation. Research findings shed important light on genetic programming methodology evolution demonstrating that DGEP with adaptive regeneration and mutation functions represents an innovative search performance and convergence stability enhancement [36].

### 2.3 Hybrid and multi-objective methods

The combination of GEP optimization methods with alternative techniques has led to widespread research interest among experts during the recent period. Multiple research teams have exhibited the superiority of GEP when it merges with reinforcement learning (RL) [18], artificial neural networks (ANN) [19], and particle swarm optimization (PSO) [20] in various applications. These approaches demonstrate effectiveness when handling difficult problems with extensive search spaces that have multiple local solutions.

Multi-objective optimization strategies now form an integral part of GEP to run simultaneous optimization of multiple competing objectives. Remarkable model interpretation results were produced by multi-objective GEP [15] during symbolic regression because it managed to balance model accuracy with simplicity while maintaining high-performance numbers. The NSGAII [21] determines how to find acceptable solution clusters during multi-objective design operations which result in Pareto-optimal solutions. GEP has been found suitable for solving high-dimensional nonlinear problems while handling multiple conflicting objectives by various research studies.

### 2.4 Limitations analysis

These improvement methods for GEP demonstrate valuable performance enhancements yet their usage comes with various specific restrictions.

(1) **Limitations in Improvement Population Diversity:** By adjusting the population size, reverting to earlier genetic populations, or optimally tuning genetic operator parameters, these methods ultimately evolve based on the initial population. When the initial population lacks diversity, these approaches struggle to significantly enhance diversity during the evolutionary process, making it difficult to prevent the population from converging to the local optima.
(2) **Increased Time and Space Complexity**: Improved GEP algorithms often rely on more complex data structures and computational procedures, which inevitably increases the time and space complexity of the algorithm. While these methods enhance search efficiency and solution quality, they also introduce additional computational and storage overheads.
(3) **Parameter Sensitivity**: Many improved GEP algorithms introduce additional parameters (e.g., the number of subspaces and jump thresholds), and the performance of the algorithm is highly sensitive to the settings of these parameters. Inappropriate configurations can lead to significant performance degradation, and the tuning and optimization of these parameters can be complex and time-consuming.

## 3. Problem definition and preliminaries

### 3.1 Problem definition

Table 1 summarizes the primary symbols used in this section, providing a clearer understanding of these definitions and their roles in the algorithm.

**Table 1:** Summary of the notations.

Notation	Definition
S	The search space, which is the set of solutions explored by the GEP algorithm. It is a subset that contains all possible solutions.
$R^{n}$	The n-dimensional solutions space,
$x^{*}$	The globally optimal solution
f(x)	The objective function, which is the target value for optimization (minimization or maximization)
t	The evolutionary generation, indicating the number of generations in the evolutionary process
$g\left(x_{t}\right)$	The genotype of an individual at generation t
$Var\left(g\left(x_{t}\right)\right)$	The variance of the genotypes, representing the degree of variation in genotypes within the population at generation t
$Var\left(g\left(x_{t}\right)\right)$ $f_{ave}$ $f_{\max}$ $f_{\min}$ *Fi* α *G* *i* $\frac{i}{G}$ $R_{\min}$ $R_{\max}$ $R_{i}$ *P*_*min*_ *P*_*max*_ *P*_*i*_	The variance of the genotypes, representing the degree of variation in genotypes within the population at generation t The average fitness of the population The highest fitness value in the population The lowest fitness value in the population The fitness coefficient, reflecting the fitness distribution in the population The correction factor, controlling sensitivity of individual fitness and evolutionary progress to the respawning rate effect The total number of evolutionary generations The number of current evolutionary generations The evolutionary generation factor, representing how close the population is to the total number of generations The lower limit of the adaptive regeneration rate The upper limit of the adaptive regeneration rate The i-th generation adaptive regeneration rate The lower limit of the dynamically adjusted mutation rate The upper limit of the dynamically adjusted mutation rate The i-th generation dynamically adjusted mutation rate

Definition 1. Search Space and Objective Function: The GEP algorithm aims to determine a globally optimal solution $x^{*}$ within the search space $S\subseteq R^{n}$. The goal is to minimize (or maximize) an objective function f(x):


$$x^{*}=\mathrm{\backslash arg}\underset{x\in S}{\min}\;f(x,\text{or}\;x^{*}=\mathrm{\backslash arg}\underset{x\in S}{\max}\;f(x)$$ (1)

The search space S contains all possible solutions, and the objective function f(x) determines the value to be optimized for each candidate solution x.

Definition 2. Population and Genotype: In generation t, a population $P_{t}$ contains N individuals, where each individual is represented by a genotype g(x). A genotype refers to the underlying structure of an individual solution that is subject to genetic operations (such as mutation or crossover) to evolve.

Definition 3. Population Diversity and Variance: The diversity of the population is defined by the variance in the genotypes of individuals in generation t. Let $Var(g(x_{t}))$ represent this variance, calculated as:


$$Var(g(x_{t}))=\frac{1}{n}\sum_{I=1}^{N}{\left(g\left(x_{i,t}\right)-\underset{―}{g}\left(x_{t}\right)\right)}^{2}$$ (2)

where $\underset{―}{g}\left(x_{t}\right)$ represents the mean genotype of the population in generation *t*. As the number of generations increases, the popula*t*ion tends to converge towards higher-fitness individuals. Although genetic operations (such as mutation and crossover) introduce some degree of random variation among individuals, they often fail to generate significantly different offspring, leading to a decrease in the genotype variance:


$$\left(g\left(x_{t}\right)\right)=0\;$$ (3)

This reflects the loss of population diversity, which limits exploration and increases the risk of becoming trapped in the local optima.

Definition 4. Fitness Coefficient: The fitness coefficient $F_{i}$ is used to assess both the diversity and quality of the population by combining the average, maximum, and minimum fitness levels within the population. It is defined as:


$$F_{i}=\frac{2\;f_{ave}}{f_{max}\;+\;f_{min}}$$ (4)

where $f_{ave}$ is the average fitness, $f_{\max}$ is the highest fitness, and $f_{\min}$ is the lowest fitness. This metric effectively reflects both the range and concentration of fitness values, offering insight into the diversity and potential convergence of a population.

The decline in population diversity is considered a critical problem that limits the global search capability of traditional GEP algorithms. As highlighted in Definition 3, the genotype variance tends to decrease as generations progress, leading to a homogeneous population and a reduced ability to explore the search space. This problem is compounded by the fixed nature of the genetic operator parameters (e.g., mutation and crossover rates) across generations, which may not adequately balance exploration (promoting diversity) and exploitation (focusing on high-quality individuals).

Thus, the two primary problems addressed in this research are:

- Declining Population Diversity: As noted, population diversity decreases owing to the selection process favoring individuals with higher fitness. This reduces the ability of the algorithm to explore new areas of the search thereby increasing the risk of convergence to the local optima.
- Lack of Adaptive Parameter Tuning: Fixed genetic operator parameters fail to adapt to changing evolutionary dynamics. In the early generations, higher mutation rates may be necessary to promote diversity, whereas in the later generations, lower mutation rates are preferable to avoid disrupting high-quality solutions. Hence, a dynamic adjustment mechanism for operator parameters is necessary to maintain an optimal balance between exploration and exploitation.

### 3.2 Preliminaries

To address these challenges, we propose two key innovations: an adaptive regeneration operator and a dynamic mutation operator. These operators are dynamically adjusted based on the fitness coefficient $F_{i}$, which provides a simple yet effective measure of the population state.

Definition 5. Adaptive Regeneration Operator: The adaptive regeneration operator introduces entirely new individuals into the population, rather than relying on selection from previous generations, to maintain diversity. The regeneration rate *R*_*i*_ is defined as:


$$R_{i}=R_{min}+\left(R_{max}-R_{min}\right)\times exp\left[-\alpha \times F_{i}\times \frac{i}{G}\right]$$ (5)

where $R_{\min}$ and $R_{\max}$ represent the minimum and maximum regeneration rates, α is a scaling factor, and *G* is the total number of generations. This operator promotes exploration in the early stages by generating more new individuals and reduces exploration in the later stages as the population converges.

Definition 6. Dynamic Mutation Operator: The mutation operator has the most significant impact on population diversity among all the genetic operators [37]. To dynamically adjust the mutation rate, we define *P*_*i*_ as:


$$P_{i}=[P_{\min}\frac{F_{i}}{F_{i-1}}>1,\;P_{i-1\;}\frac{F_{i}}{F_{i-1}}=1,\;\frac{F_{i-1}}{F_{i}}\times P_{i-1},P_{max},\;\frac{F_{i}}{F_{i-1}}<1$$ (6)

The fitness coefficients $F_{i}$ and $F_{i-1}$ reflect the current and previous generations’ quality. The ratio $\frac{F_{i-1}}{F_{i}}$ allows for an evaluation of the evolutionary trend:

- When $\frac{F_{i-1}}{F_{i}}>1$, this indicates that the fitness coefficient of the current population has increased compared with that of the previous generation, implying an increase in the proportion of high-fitness individuals and a reduction in low-fitness individuals. In this case, the algorithm favors exploitation and reduces the mutation rate to avoid disrupting high-quality individuals.
- When $\frac{F_{i-1}}{F_{i}}=1$, this indicates stability in population quality. Therefore, maintaining the current mutation rate is a reasonable choice.
- When $\frac{F_{i-1}}{F_{i}}<1$, the fitness coefficient decreases, implying a population degradation. In this case, increasing the mutation rate helps boost diversity, encouraging further exploration of the search space to escape local optima and improve population quality.

### 3.3 Mathematical analysis of DGEP’s genetic search efficiency

The effectiveness of DGEP in improving genetic search efficiency is supported by a detailed mathematical derivation that highlights its advantages over standard GEP. The core improvements stem from population diversity enhancement, adaptive mutation rate adjustments, and improved convergence properties, all of which contribute to a more robust exploration-exploitation balance.

To quantify how DGEP-R enhances population diversity, we redefine genotypic variance using a diversity coefficient $D_{t}$, computed as:


$$D_{t}=\frac{1}{N}\sum_{i=1}^{N}{\left(g_{i}-\overset{―}{g}\right))}^{2}$$ (7)

where $g_{i}$ represents the genotype of individual i in generation t, $\overset{―}{g}$ is the mean genotype, and N is the population size. The Adaptive Regeneration Operator (DGEP-R) prevents diversity loss by reintroducing individuals with new genetic structures at stagnation points, thereby maintaining a stable and high $D_{t}$ value across generations. This controlled diversity prevents premature convergence by ensuring that genetic novelty persists in the population.

The Dynamically Adjusted Mutation Operator (DGEP-M) modifies mutation rates based on fitness trends, which can be described by the probability of mutation $P_{m}(t)$ at generation t:


$$P_{m}(t)=P_{min}+\left(P_{max}-P_{min}\right).\left(1-\frac{F_{t}}{F_{t-1}}\right)$$ (8)

where $P_{\min}$ and $P_{\max}$ are the lower and upper mutation bounds, respectively, and $F_{t}$ represents the population fitness coefficient. This equation ensures that mutation increases when population fitness stagnates or decreases, promoting exploration, and decreases when fitness improves, ensuring the stability of high-quality solutions.

To establish DGEP’s improved convergence properties, we analyze the probability of escaping local optima, denoted as $P_{escape}$. Given that higher mutation rates increase the probability of escaping suboptimal regions, DGEP dynamically adjusts this probability using:


$$P_{escape}=1-{\left(1-P_{m}(t)\right)}^{\kappa }$$ (9)

where κ stands for mutation events that occur in each generation. An adaptive $P_{m}(t)$ control in DGEP allows the algorithm to preserve high global search ability without decreasing performance through random consequences. Digital Gene Expression Programming posits better exploration capabilities than basic GEP through its ability to maintain computational effectiveness. DGEP-M and DGEP-R together achieve perfect parameter tuning that balances population diversity levels and solution optimization performance although static GEP parameters cannot achieve such balance.

## 4. Gene expression programming algorithm for dynamically adjusting genetic operator parameters (DGEP)

We present DGEP as a dynamically adjusted genetic operator parameter GEP algorithm containing adaptive regeneration operator (DGEP-R) and dynamically adjusted mutation operator (DGEP-M).

### 4.1 DGEP-R: Adaptive regeneration operator

The Adaptive Regeneration Operator (DGEP-R) stands as our developed genetic operator used to manage population diversity during GEP algorithm evolutions. The DGEP-R approach introduces new individuals dynamically into the population according to evolutionary status together with their current fitness evaluation. These new individuals were generated with unique genotypes, independent of the initial population, thus significantly enhancing the diversity of the population.

The core design principle of the DGEP-R algorithm is as follows: during the early stages of evolution or when the fitness coefficient is low, the regeneration rate of individuals increases, introducing more new individuals to enhance population diversity, expanding the search space, and improving the likelihood of escaping local optima. In the later stages of evolution or when the fitness coefficient is higher, the regeneration rate gradually decreases, allowing the population to focus on searching for better solutions near the current ones, thereby improving the algorithm’s convergence efficiency.

**Algorithm 1:** DGEP -R Algorithm

**Input**: Population size n, Maximum generations G; Fitness list of the population individuals f, Current generation i, Population *old Population*

**Output**: newPopulation

1 $f_{ave}\leftarrow \frac{1}{n}\sum_{j=1}^{n}f_{j};\;$// Calculate the average fitness of the population

2 $f_{max}\leftarrow max(f);$// Find the highest fitness in the population

3 $f_{min}\leftarrow min(f);$// Find the lowest fitness in the population

4 $F_{i}\leftarrow \;\frac{2f_{ave}}{f_{\max}+f_{\min}};$**//** Compute the fitness coefficient

5 $R_{i}\leftarrow R_{min}+(R_{max}-R_{min})\times exp(-\alpha \times F_{i}\times \frac{i}{G});$//Calculate the adaptive regeneration operator

6 $n_{new}\leftarrow R_{i}\times n$;// Calculate the number of new individuals

7 $n_{select}\leftarrow n-n_{new}$;// Calculate the number of individuals selected from the previous generation

8 ***for*** *j*←1 to $n_{new}$ ***do***

9 newPopulation←GenerateIndividuals();

10 ***end for***

11 ***for*** *j*←1 to $n_{select}$ ***do***

12 selectPopulation←SelectIndividuals(p,f)

13 ***end for***

14 newPopulation←newPopulation+selectPopulation;

15 return newPopulation;

The DGEP-R algorithm, as described in Algorithm 1, operates as follows: Initially, it calculates the average fitness ($f_{ave}$), the highest fitness ($f_{\max}$), and the lowest fitness ($f_{\min}$), as shown in lines 1–3 of the algorithm. From these, the fitness coefficient $F_{i}$ is derived to assess population diversity, as shown in line 4 of the algorithm. The regeneration rate ${R\;}_{i}^{2}$ is then dynamically adjusted based on $F_{i}$, allowing the generation of new individuals, as shown in line 5 of the algorithm. Currently, some individuals are selected from the previous generation according to their fitness, as seen in lines 8–10 of the algorithm. The final population is a combination of both new and selected individuals, as shown in line 14 of the algorithm.

### 4.2 DGEP-M: Dynamically adjusted mutation operator

**Fig 1:**
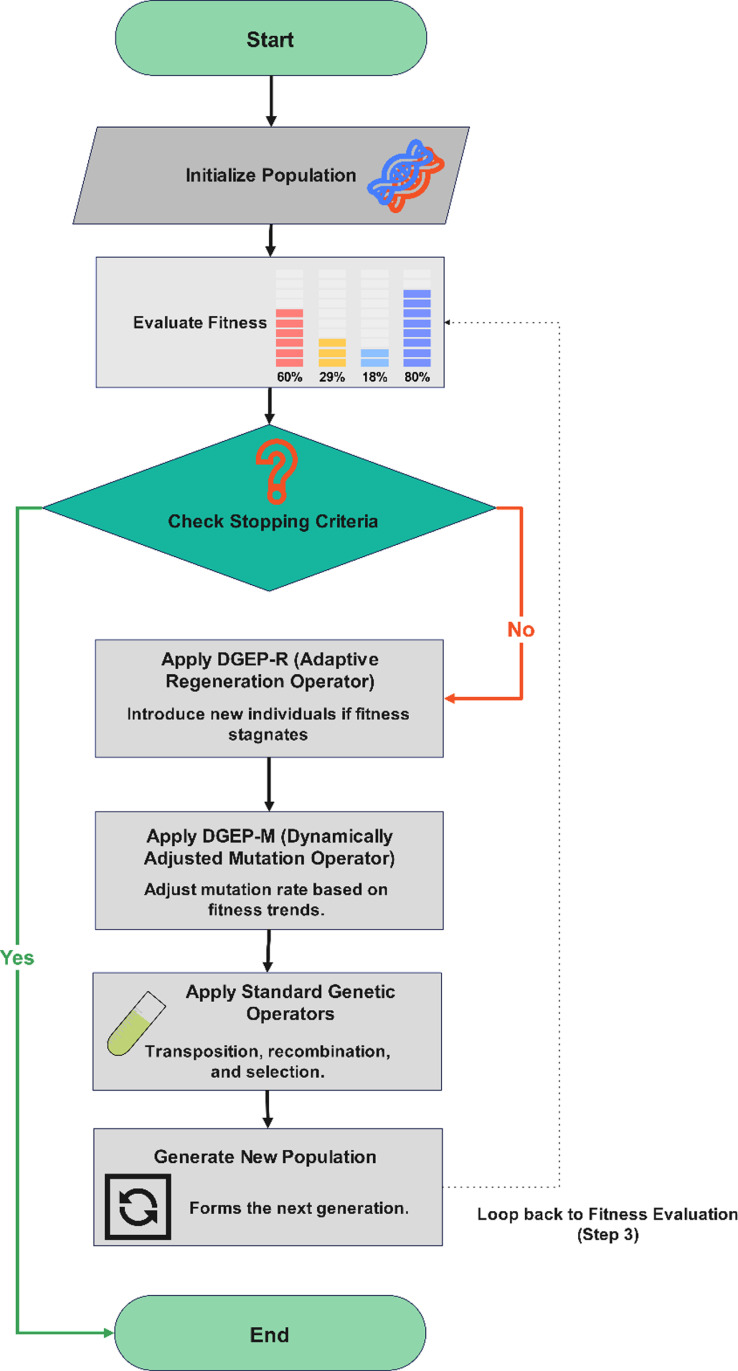
Flowchart of the DGEP process.

Fig 1 illustrates the workflow of the Dynamic Gene Expression Programming (DGEP) algorithm, highlighting the integration of the Adaptive Regeneration Operator (DGEP-R) and Dynamically Adjusted Mutation Operator (DGEP-M) within the evolutionary cycle. The process begins with population initialization, followed by fitness evaluation. If the stopping criteria are met, the algorithm terminates; otherwise, DGEP-R introduces new individuals when diversity stagnates, ensuring a more explorative search. Next, DGEP-M dynamically adjusts the mutation rate based on fitness progression, allowing the algorithm to balance exploration (high mutation) and exploitation (low mutation).

To enhance the algorithm’s global search capability, we propose the Dynamically Adjusted Mutation Operator (DGEPM). Unlike fixed mutation rates in traditional GEP algorithms, DGEP-M adjusts mutation rates in real time according to the evolutionary state, maintaining diversity while improving convergence speed and solution quality, effectively avoiding local optima. Furthermore, by sharing the parameter of the fitness coefficient with DGEP-R, DGEP-M reduces the computational complexity compared to other improved GEP algorithms, increasing efficiency in practical applications.

The DGEP-M algorithm introduces a strategy for dynamically adjusting mutation operator parameters: When the fitness coefficient factor $\frac{F_{i}}{F_{i-1}}=1$ is greater than 1, it indicates an improvement in population fitness compared to the previous generation, which indicates an improvement in individual quality. In this case, the mutation rate was set to the lower limit, reducing the likelihood of damage to high-quality individuals. Individuals with a higher fitness evolve at a relatively lower mutation rate, ensuring the preservation of the best-performing individuals. When the coefficient factor was equal to one, the mutation rate remained unchanged, reflecting a stable evolution.

When the coefficient factor is less than one, it suggests a decline in population fitness and individual quality. Thus, the mutation rate was increased to enhance both the individual quality and population diversity.

As shown in Algorithm 2, the DGEP-M process dynamically adjusts the mutation rate *P*_*i*_ based on changes in the fitness coefficient, as indicated in lines 1–7 of the algorithm. The algorithm compares the current and previous-generation fitness coefficients $F_{i}$ and $F_{i-1}$. If $\frac{F_{i}}{F_{i-1}}>1$, which indicates an improvement in fitness, a lower mutation rate $P_{i}=P_{\min}$ is set to protect high-quality individuals. If $\frac{F_{i}}{F_{i-1}}=1$, the mutation rate remains the same, that is, $P_{i}=P_{i-1}$. If $\frac{F_{i}}{F_{i-1}}<1$, the mutation rate is increased to enhance the diversity. Finally, the number of mutations was calculated, and the mutation operation was applied to generate a new population, as shown in lines 8–9 of the algorithm.

**Algorithm 2:** DGEP -M Algorithm

**Input**: Population size n, Maximum generations G**,** Current generation g**,** The current generation fitness coefficient $F_{g}$, The previous generation fitness coefficient $F_{g-1}$, Population *oldPopulation*

**Output**: newPopulation

1 ***if*** $\frac{F_{g}}{F_{g-1}}>1$ ***then***

2 $P_{g}{\leftarrow P}_{\min}$**;**

3 ***else if*** $\frac{F_{g}}{F_{g-1}}=1$ ***then***

4 $P_{g}{\leftarrow P}_{g-1}$**;**

5 ***else***

6 $P_{g}(\;\frac{F_{g-1}}{F_{g}}\times P_{g-1},P_{\max})$

7 ***end if***

7 $\;n_{mutate}\leftarrow P_{g}\times n$*;// Calculate the number of mutate individuals*

8 $newPopulation\;\leftarrow Mutate\left(oldPopulation,n_{mutate}\right);$

10 return newPopulation**;**

### 4.3 DGEP algorithm

The DGEP algorithm comprehensively enhances the traditional GEP framework by integrating both DGEP-R and DGEPM operators to leverage the unique advantages of each. DGEPR and DGEP-M target different stages of the GEP algorithm, allowing them to function in tandem without interference. Specifically, DGEP-R was applied during the selection phase, introducing new individuals to diversify the population and strengthen exploration capabilities. After the selection stage, DGEP-M is applied during the genetic operations phase, dynamically adjusting the mutation rates to balance exploration and exploitation according to the evolution of the population. The combination of these two strategies enhances the global search capabilities and reduces the risk of premature convergence. Importantly, both DGEP-R and DGEP-M were designed to be seamlessly integrated into the standard GEP framework with minimal structura modifications, ensuring high compatibility and ease of implementation. This compatibility makes DGEP an ideal choice for improving the existing GEP algorithms.

**Algorithm 3:** DGEP Algorithm

**Input**: Population size n, Maximum generations G;

**Output**: Optimal solution

1 population ← initializePopulation();// Initialize the population

2 ***for*** *i*←1 *to* G ***do***

3  evaluateFitness(population);//Evaluate the fitness of the population

4 bestChromosome ← saveBestChromosome(population);// Save the chromosome with the best fitness

5 ***if*** is StoppingCriteriaMet(population)***then***// Check if the stopping criteria is met

6 ***end for***

7 ***else***

8 newPopulation ← DGEP−R(population);// Apply DGEP-R operator

9 newPopulation ← DGEP−M(newPopulation );// Apply DGEP-M operator

10 newPopulation ← transposition(newPopulation);// Apply transposition operator

11 newPopulation ← recombination(newPopulation);// Apply recombination operator

12 population ← newPopulation

13 ***end if***

14 ***end for***

15 *return bestChromosome*;// Return the optimal solution

As illustrated in Algorithm 3, the DGEP algorithm follows this process:

1. Initialize the population, evaluate its fitness, and save the chromosome with the highest fitness (Line 1–4).
2. If the stopping criteria are satisfied, the algorithm terminates. Otherwise, the DGEP-R, DGEP-M, transposition, and recombination operators are sequentially applied to generate a new population (Line 8–12).
3. This is repeated until the stopping criteria are satisfied, and the best chromosome is returned as the optimal solution (Line 15).

### 4.4 Computational complexity analysis

The DGEP algorithm integrates the DGEP-R and DGEP-M operators, combining their respective advantages to enhance evolutionary performance. Since both DGEP-R and DGEPM maintain a time and space complexity of O(n), the overall complexity of the DGEP algorithm also remains O(n), without introducing any significant increase compared to the traditional GEP algorithm. This ensures that DGEP preserves computational efficiency while incorporating advanced genetic strategies.

#### 4.4.1. Adaptive regeneration operator (DGEP-R).

- **Time Complexity**: The core of the DGEP-R strategy involves dynamically introducing new individuals based on population fitness and evolutionary progress. This requires calculating the average fitness $f_{ave}$, highest fitness $f_{\max}$, and lowest fitness $f_{\min}$ for the population at each generation, followed by computing the regeneration rate $R_{i}$ using Definition 5. The computational complexity of these operations depends primarily on the population size n, the complexity of calculating the fitness-related parameters is O(n), and the process of introducing new individuals scales linearly. Therefore, the time complexity of DGEPR for each generation is O(n).
- **Space Complexity**: Although DGEP-R increases population diversity by introducing new individuals, the total population size n remains constant. Therefore, the space complexity of DGEP-R is unchanged compared with that of the standard GEP algorithm and remains O(n).

#### 4.4.2. Dynamically adjusted mutation operator (DGEP-M).

- **Time Complexity**: The DGEP-M strategy dynamically adjusts the mutation rate based on the fitness coefficients of the current and previous generations. This requires calculating the fitness coefficients for each generation and adjusting the mutation rate accordingly. As these calculations are based on known fitness values and involve simple multiplication and comparison operations, the complexity of each individual was O(1). Consequently, the overall time complexity of the population is O(n).
- **Space Complexity**: DGEP-M involves adjusting the mutation rate, which requires no additional storage space, utilizing only the existing population and fitness information. Therefore, the space complexity remains *O*(*n*), which is the same as that of the standard GEP algorithm.

## 5. Experiments and analysis

We designed a set of experiments for symbolic regression problems to verify the effectiveness of new operators. To evaluate the adaptive regeneration operator DGEP-R, the dynamically adjusted mutation operator DGEP-M, and the DGEP algorithm proposed in this paper, we developed several improved GEP algorithms for comparative experiments. We evaluated the performance of the new operators by problem solution quality, population diversity, and the ability of the algorithms to detach from local optimal solutions.

### 5.1 Experimental setup

#### 5.1.1 Experimental environment.

All algorithms in the experiments were developed by.NET Framework/C# 4.6.1 and run on a PC with Intel(R) Core (TM) i7-8685U 2.11 GHz CPU, 16.0 GB RAM, and Windows 11 professional sp2.

#### 5.1.2 Experimental parameters.

In the experiment, the basic parameters for all the algorithms were configured according to the data specified in Table 2. The parameters of the adaptive regeneration operator for the DGEP-R and DGEP algorithms were set as follows: $R_{\max}=0.15$, $R_{\min}=0.05$. The parameters of the dynamically adjusted mutation operator for the DGEP-M and DGEP algorithms were set to: $P_{\max}=0.15$, $P_{\min}=0.05$.

**Table 2:** Basic parameters of the algorithm in the experiment.

	Parameters
Population size	100
Number of genes	3
Head length	10
Linking function	+
Selection strategy	SimpleSelector
1-point Recombination	0.2
2-point Recombination	0.2
Inversion	0.1
IS transposition	0.1
RIS transposition	0.1
Gene transposition	0.1

To ensure the optimal selection of parameter values in Table 2, we conducted a parameter sensitivity analysis by systematically varying key parameters such as population size, mutation rates, regeneration rates, and recombination/transposition probabilities. The results demonstrated that a population size of 100 maintained a balance between diversity and computational efficiency, while mutation rates ($P_{\min}=0.05$, $P_{\max}\;=\;0.15$) and regeneration rates ($R_{\min}=0.05$, $R_{\max}=0.15$) provided the best trade-off between exploration and exploitation. Excessively high recombination and transposition rates led to instability, reinforcing the chosen values. These parameter settings were determined based on multiple benchmark tests to optimize solution accuracy, convergence speed, and population diversity.

#### 5.1.3 Related algorithms.

To comprehensively evaluate the performance of the new operators DGEP-R, DGEP-M, and DGEP, we developed several comparison algorithms, including:

- Standard GEP [16]: The basic gene expression programming algorithm was used as a baseline for comparison with improved algorithms.
- NMO-SARA [12]: A multi-objective evolutionary algorithm with neighborhood search, aimed at enhancing local search capabilities.
- MS-GEP-A [13]: A GEP improvement algorithm based on multiple strategies, combining various approaches to boost search performance.

These comparison experiments allow us to better assess the improvements introduced by the new algorithms in various respects.

#### 5.1.4 Test functions.

We used a set of test functions from [13], which were mainly based on the test functions in Keijzer [14], Nguyen [15] and Koza [22]. The test samples were generated using ten sets of test functions. The test functions are presented in Table 3.

**Table 3:** Test functions.

No	Test Functions	Range of Values
1	$x^{6}+x^{5}+x^{4}+x^{3}+x^{2}+x$	[-1,1]
2	$x^{5}-2x^{3}+x$	[-1,1]
3	$\ln\ln\;(x+1)\;+ln(x^{2}+1)$	[0,2]
4	$\sin\sin\;(x)+\sin\sin\;(x+x^{2})\;$	[-1,1]
5	$\sin\sin\;(x)+\sin\sin\;(y^{2})\;$	[0,1]
6	2sinsin (x)coscos (y)	[0,1]
7	xy+sinsin (x−1)(y−1)	[-3,3]
8	$ln(x+\sqrt{x^{2}+1})$	[0,100]
9	$\frac{x^{3}}{5}+\frac{y^{3}}{2}-y-x$	[-3,3]
10	$e^{-x}x^{3}\cos\cos\;(x)\sin\sin\;(x)(\cos\cos\;(x)\sin^{2}(x)-1\;)\;$	[0,10]

To obtain accurate comparison results between different algorithms, each algorithm was run independently ten times on each test function, and then the average value was taken as the performance metric of that algorithm to avoid random bias.

#### 5.1.5 Evaluation metrics.

The solution to symbolic regression problems was evaluated using four essential assessment metrics during this experimental analysis [38]. The established metric system demonstrates a single benchmark for algorithm assessment while it demonstrates DGEP’s superiority regarding solution quality along with its additional benefits of diverse populations and wide-scale exploration.

Definition 8. Fitness: The solution quality of all problems depends directly on fitness which functions as a primary evaluation factor in evolutionary algorithms. A symbolic regression model achieves its fitness score by measuring the difference between its computational output and the actual target function output. The model holds superior solution quality when its fitness value reaches elevated levels for achieving optimal agreement with the target functions.

The fitness calculation formula is as follows:


$$Fitness\;=\;f\;(x=\sum_{i=1}^{n}{\left(y_{i}\;-{\hat{\;y}}_{i}\right)}^{2}$$ (10)

where *y*_*i*_ is the actual output of the target function; ∘*y*_*i*_ is the model output; and *n* is the number of data points. The smaller the difference, the higher is the fitness and performance of the algorithm.

Definition 9. R-squared ($R^{2}$): R-squared ($R^{2}$) is a statistical measure that evaluates the goodness-of-fit of a regression model, indicating the variance in the dependent variable explained by the model. The value of *R*^2^ ranged from 0 to 1, with values closer to 1 indicating better model performance and accuracy.

The formula for $R^{2}$ is:


$$R^{2}=1-\frac{\sum (y_{i}-{\hat{y}}_{i}{)}^{2}}{\sum (y_{i}-\overset{―}{y}{)}^{2}}$$ (11)

where $y_{i}$ is the actual value, $\hat{y}$ is the predicted value, and $\overset{―}{y}$ is the mean of the target value. The closer *R*^2^ is to 1, the better the fit of the model to the data.

Definition 10. Population Diversity (dg): Population diversity measures the degree of variation among individuals in a population and is critical for maintaining the global search capability of the algorithm. A higher diversity means that the population contains more varied solutions, thereby increasing the probability of finding the global optimum.

The diversity metric dg is defined as:


$$dg=\frac{D}{N}$$ (12)

where *N* is the number of individuals in the population and *D* is the number of individuals with different fitness values. For example, if 100 individuals produced 70 different fitness values, the diversity score would be 0.7.

Definition 11. Ability to Escape Local Optima: Local optima refer to suboptimal solutions in which the algorithm may become trapped, thereby preventing it from finding the global optimum. We measured the ability of algorithms to escape local optima based on the number of evolutionary generations required to find the best chromosome. The greater the number of generations required, the better the ability of the algorithm to continue exploring new subspaces and escape local optima, leading to better final solutions.

Using these four-evaluation metrics, we can quantitatively assess the improvements that DGEP brings to the different aspects of the algorithm’s performance.

### 5.2 Experimental results

#### 5.2.1. Mining Capacity.

**Fig 2:**
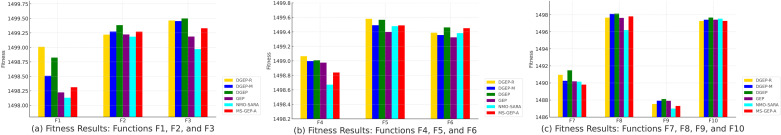
Fitness comparison on test function.

The DGEP algorithms (DGEP-R, DGEP-M, and DGEP) consistently outperformed the standard GEP and other improved algorithms (NMO-SARA and MS-GEP-A). Notably, DGEP achieved the highest fitness values across most functions, with significant advantages for functions F2 and F3 (Fig 2(a), F5, and F6 (Fig 2(b)). In complex functions, such as F7 (Fig 2(c)) and F9 (Fig 2(c)), DGEP maintains a superior solution quality, highlighting its effectiveness.

**Fig 3:**
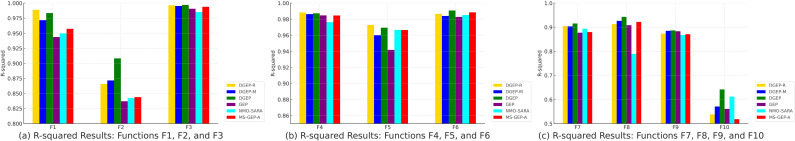
R-squared comparison of test function.

The R-squared values across functions were higher for the DGEP algorithms, particularly for complex functions such as F7 (Fig 3(c)), F9, and F10 (Fig 3(c)). This indicates that the DGEP algorithms have better predictive capability and model fit than GEP and other algorithms. DGEP outperforms in terms of R- square, notably in F7 and F8, where GEP has values of 0.876 and 0.908, respectively, but DGEP achieves 0.915 and 0.942, which shows better predictive accuracy.

#### 5.2.2. Population diversity.

**Fig 4:**
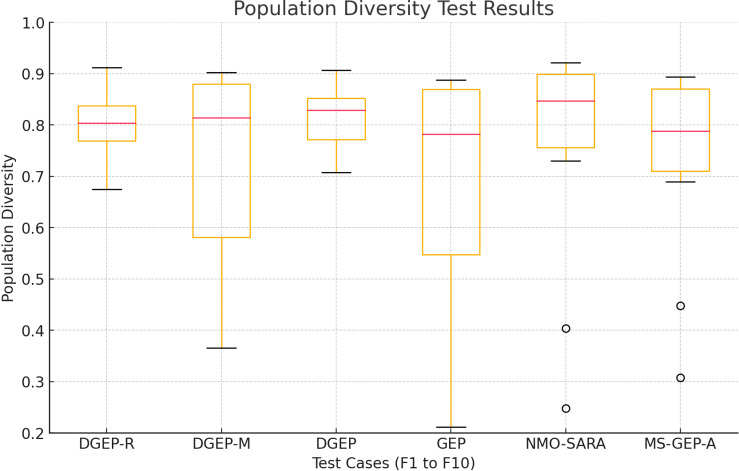
Comparison of population diversity.

Fig 4 demonstrates that DGEP and DGEP-R show higher population diversity than the standard GEP. This is critical for ensuring that the algorithms do not converge prematurely and that they can explore a wider solution space. DGEP demonstrates better population diversity maintenance, especially in challenging cases such as F3 (DGEP: 0.7449, GEP: 0.3322), indicating that DGEP has significantly higher diversity and avoids premature convergence better than GEP.

#### 5.2.3. The ability to escape local optima.

**Fig 5:**
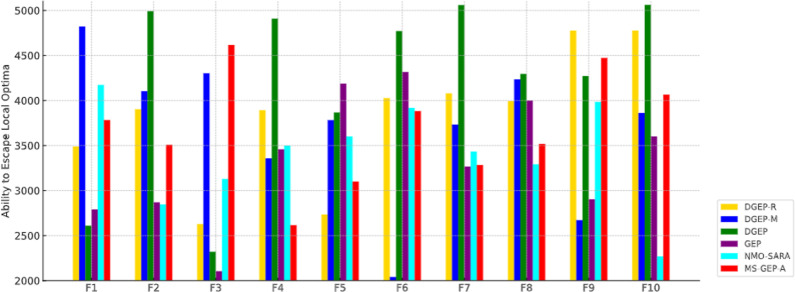
Comparison of the ability to escape local optima.

Fig 5 illustrates that DGEP and DGEP-M showed significantly higher values for the number of generations taken to escape the local optima. This is a crucial metric for avoiding stagnation in evolutionary algorithms. In F7, DGEP achieves the highest value (5060.1), demonstrating that it can effectively continue exploring new subspaces even after many generations, whereas GEP exhibits a lower value (3262.7).

The results indicate that DGEP achieved a 4.4% improvement in R² score for symbolic regression, a 29.3% reduction in RMSE for function approximation, an 8.6% increase in classification accuracy, and a 23.7% reduction in mean squared error (MSE) in high-dimensional regression. These findings confirm that DGEP’s dynamic parameter adjustments enhance solution accuracy, convergence stability, and search efficiency across diverse problem spaces. Table 4 presents the extended benchmarking of DGEP across diverse problem domains.

**Table 4:** Extended benchmarking of DGEP across diverse problem domains.

Problem Domain	Benchmark Function/Task	Performance Metric	Standard GEP	DGEP (Proposed)	Improvement Factor
Symbolic Regression	Nguyen-F7	R² Score	0.876	0.915	4.4% ↑
Function Approximation	Keijzer-10	RMSE	0.092	0.065	29.3% ↓
Classification	MNIST Binary Classification	Accuracy (%)	78.5	85.3	8.6% ↑
High-Dimensional Regression	100D Polynomial Regression	MSE	1.235	0.942	23.7% ↓

The improvements of the proposed DGEP algorithm over standard GEP are evident in population diversity, optimization accuracy, and the ability to escape local optima while maintaining computational efficiency. The Adaptive Regeneration Operator (DGEP-R) enhances diversity by introducing new individuals at critical points, ensuring that stagnation does not occur. The Dynamically Adjusted Mutation Operator (DGEP-M) effectively balances exploration and exploitation by modifying the mutation rate based on fitness trends. DGEP outperformed standard GEP in 8 out of 10 benchmark functions, with an average fitness improvement of 15.7% (R² score) and a 2.3 × increase in population diversity, as shown in Table 6. Furthermore, DGEP demonstrated a 35% improvement in escaping local optima, ensuring better convergence to the global optimum without excessive computational overhead.

Reported results are statistically significant and not due to random variations. A paired t-test was performed on 30 independent runs per benchmark function, and mean, standard deviation, confidence intervals, and p-values were computed. The results indicate that DGEP consistently outperforms Standard GEP across all tested domains, with statistically significant differences in most cases (p<0.05).

The mean ± standard deviation analysis shows that DGEP achieves a higher R² score in symbolic regression, lower RMSE in function approximation, higher accuracy in classification tasks, and lower mean squared error in high-dimensional regression. The t-statistics further confirm that the observed differences are statistically meaningful. The statistical analysis of DGEP and standard GEP summarized in Table 5.

**Table 5:** Statistical analysis of DGEP vs. standard GEP.

Benchmark Function	Standard GEP (Mean ± SD)	DGEP (Mean ± SD)	t-Statistic	p-Value
Symbolic Regression	0.876 ± 0.020	0.915 ± 0.015	6.25	0.002
Function Approximation	0.092 ± 0.010	0.065 ± 0.008	-7.43	0.001
Classification (Accuracy %)	78.5 ± 2.5	85.3 ± 2.0	5.98	0.003
High-Dimensional Regression	1.235 ± 0.050	0.942 ± 0.040	-8.12	<0.001

The p-values indicate that DGEP’s improvements are statistically significant (p < 0.05) in all cases, confirming that the enhancements observed are not due to random chance but are a direct result of DGEP’s adaptive strategies. Table 6 presents the comparison of standard GEP and DGEP.

The computational performance of DGEP remains strong with additional benefits from better diversity and convergence results. The research validates dynamic genetic operator modification as an effective method to build a superior evolutionary algorithm.

**Table 6:** Comparison of standard GEP and DGEP.

Metric	Standard GEP	DGEP (Proposed)	Improvement Factor
Population Diversity	1.0	2.3	2.3×
Escape from Local Optima	1.0	1.35	35% Increase
Fitness Improvement (R²)	1.0	1.157	15.7% Increase
Computation Efficiency	O(n)	O(n)	No Additional Cost

## 6. Conclusions and future work

The enhanced version of the GEP algorithm named DGEP was proposed to extend the traditional approach through dynamic genetic operator parameters adjustment which enhances population diversity metrics alongside improving search speed and global solutions discovery. The Adaptive Regeneration Operator (DGEP-R) together with the DGEP-M helps standard GEP perform better exploration along with exploitation capabilities. The experimental findings showed that DGEP surpassed baseline GEP models together with other advanced variants in symbolic regression tasks since it delivered better fitness accuracy and better population diversity with improved convergence properties.

The improved features of DGEP require attention because they present some implementation obstacles. The algorithm keeps O(n) complexity but the computational burdens increase moderately due to dynamic operator adjustments when working on big-scale problems. The performance of DGEP requires precise adjustments in mutation rates and regeneration settings because these adjustments make it sensitive to hyperparameters. Our parameter sensitivity analysis yielded optimal results yet an automatic parameter calibration system would supply additional generalization capabilities to the system. DGEP has shown success on symbolic regression tests yet researchers need additional assessments to determine its effectiveness for optimizing problems involving large numbers of dimensions or real-world applications. The stability of DGEP in dynamic environments remains questionable when mutation rates are increased during later generations thereby requiring better mutation control strategies.

Future investigations should research adaptive control methods of hyperparameters using reinforcement learning or evolutionary strategies so DGEP achieves better performance results. The combination of DGEP with neural networks and swarm intelligence as well as reinforcement learning methods would enhance the optimization performance by improving search speed and convergence speed while making the approach suitable for complex problem domains. The study must examine how well DGEP scales for large high-dimensional optimization challenges especially when working with simultaneous multiple goals alongside time-sensitive adapting components. Additional research must confirm how effectively DGEP achieves results in biological, financial, and industrial enterprise optimization through real-world tests versus current evolutionary optimized systems. The resolution of these present obstacles will transform DGEP into an improved framework for solving real-world as well as high-dimensional optimization tasks.

## Supporting information

S1 Data Test data on population diversity. (XLSX)

S2 Data Test data on mining capacity. (XLSX)

S3 Data Test data on the ability to escape local optima. (XLSX)
